# Climatic Trends in Hail Precipitation in France: Spatial, Altitudinal, and Temporal Variability

**DOI:** 10.1155/2013/494971

**Published:** 2013-11-02

**Authors:** Lucía Hermida, José Luis Sánchez, Laura López, Claude Berthet, Jean Dessens, Eduardo García-Ortega, Andrés Merino

**Affiliations:** ^1^Group of Atmospheric Physics, IMA, University of León, 24071 León, Spain; ^2^Anelfa, 52 rue Alfred Duméril, 31400 Toulouse, France

## Abstract

Hail precipitation is characterized by enhanced spatial and temporal variability. Association Nationale d'Etude et de Lutte contre les Fléaux Atmosphériques (ANELFA) installed hailpad networks in the Atlantic and Midi-Pyrénées regions of France. Historical data of hail variables from 1990 to 2010 were used to characterize variability. A total of 443 stations with continuous records were chosen to obtain a first approximation of areas most affected by hail. The Cressman method was selected for this purpose. It was possible to find relationships between spatial distributions of the variables, which are supported by obtained Pearson correlations. Monthly and annual trends were examined using the Mann-Kendall test for each of the total affected hailpads. There were 154 pads with a positive trend; most were located between Tarbes and Saint-Gaudens. We found 177 pads with a negative trend, which were largely south of a pine forest in Landes. The remainder of the study area showed an elevated spatial variability with no pattern, even between relatively close hailpads. A similar pattern was found in Lérida (Spain) and Southeast France. In the entire area, monthly trends were predominantly negative in June, July, and August, whereas May had a positive trend; again, however, there was no spatial pattern. There was a high concentration of hailpads with positive trend near the Pyrenees, probably owing to orographic effects, and if we apply cluster analysis with the Mann-Kendall values, the spatial variability is accentuated for stations at higher altitude.

## 1. Introduction

Hail is considered a natural risk. In Europe, this type of precipitation occurs mainly from May to September, and more commonly in continental areas [1, 2]. Thus, France is one of the European countries most affected by hail, which causes millions of Euros in losses to agriculture and property each year. The greatest risk is in southwestern France [3] and the Southern Alps [2], and it is more frequent during the warm season.

It is in these areas of high hail risk where *Association Nationale d'Etude et de Lutte contre les Fléaux Atmosphériques* (ANELFA) installed an extensive network of hailpads, which it has managed since 1987. These types of networks are usually installed in locations of frequent hail (e.g., [1, 4, 5]), since they provide objective hail measurements at low cost. The characteristics of hail in France have been thoroughly analyzed in prior studies, thanks to the available record length. Berthet et al. [6] found greater frequency and intensity of hail in the Pyrenean area, which was attributed to proximity of the mountain chains and Ebro Valley in Spain, where frequent mesoscale cyclonic systems develop during summer and cross into France over the Pyrénées [7]. 

Hailfall is a small-scale phenomenon, normally within a few square kilometers [8, 9]. The strong variability in frequency and distribution of hail is attributed to, among other things, its formation process [1]. This variability can occur on different scales, from as small as hydrometeor size to the mesoscale. Although hailpad networks provide useful information on the characteristics of precipitated hailstones, knowledge of the number of measurement points necessary to adequately characterize hail-affected areas has been a crucial problem in numerous studies. This remains an open question in need of further study. In fact, it is very common to find that data from a hailfall point are considered representative of a much larger area. Therefore, one must bear in mind that such data are used in climatological studies as well as in the calibration, validation, and verification of models [10, 11]. 

The hailpad networks of ANELFA have a high density of points, at which one can obtain objective information on hailfall characteristics. These networks provide a huge database of hail events, much larger than that typically accumulated from similar networks. Therefore, through analysis of the network data, we can enhance our knowledge of hailfall, especially concerning factors that may be involved in its variability, and even improve the ability to detect any temporal tendencies. 

In this paper, we first analyze the spatial variability of hail precipitation in southwestern France. Then, we investigate climatic variability of the number of hailpads impacted within the ANELFA network and obtain climatic trends. Then, we focus on analyzing the relationship between orography and hail variability. Finally, we obtain tendencies in other nearby areas (Ebro Valley, Spain, and Southeast France). 

## 2. Study Area and Dataset

ANELFA has historical records from four hailpad networks. The two networks with the longest records of hail precipitation measurement are the Atlantic and Midi-Pyrénées networks, in southwestern France.

The Atlantic area is nearly flat, with only a few small hills around the vineyards of Bordeaux and in the south. The Pyrénées region is mountainous and is crossed by valleys oriented northward in the central part, and eastward in the eastern half [12]. Elevations reach 3 km in the central chain [6]. The Massif Central partially extends through the regions of Languedoc-Rosellon, Rhônes-Alpes, Auvergne, Midi-Pyrénées, and Limousin.

**Figure 1:**
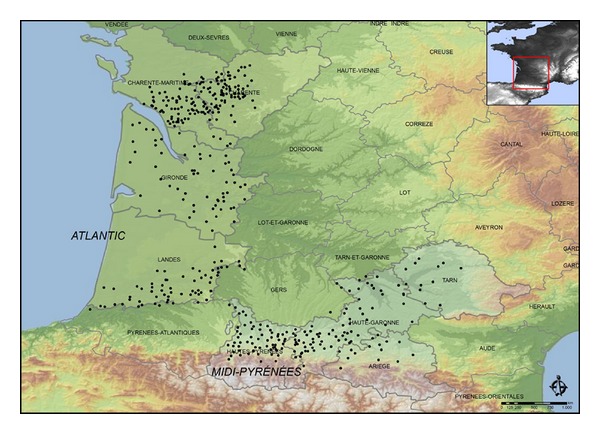
Location of the 443 ANELFA hailpad stations in continuous use between 1990 and 2010, used in the study.

The hailpad is a method of measurement that allows the study of hailfall characteristics. The hailpad material, calibration and analysis procedure have been described [8, 13]. There is historical record data from 1157 hailpads between 1990 and 2010 in France, distributed between two networks: 670 in the Atlantic and 487 in the Midi-Pyrénées areas. Nevertheless, since our objective is to analyze precipitation variability, the hailpad stations in the present database were chosen very carefully. Towards this end, we considered the station history. Many lack complete records for various reasons, one of which is that the station may have been eliminated or changed location. Station measurements may be easily affected by nonclimatic factors such as changes in instrumentation, exposure, measurement technique, different observers, or station environment. With this in mind, 443 stations were selected. Within the hailpad networks of the Atlantic and Midi-Pyrénées areas, these stations have long, continuous data records over the 21-year data collection in the experimental campaigns. Therefore, we deal with databases of hailpad stations chosen such that their data are complete and have been unaffected by change over the years. This resulted in a total of 280 hailpads in the Atlantic and 163 in the Midi-Pyrénées. These are mainly in the French departments of Charente, Charente-Maritime, Gironde, and Landes in the Atlantic area and in Haute-Garonne, Ariège, Hautes-Pyrénées, and Tarn in the Midi-Pyrénées area. There are five hailpads near the edge of the departments of Gers and Tarn-et-Garonne, and three pads in the Pyrénées-Atlantiques near the edge of Landes; these were considered part of the study area defined previously. The spatial distribution of the hailpads is dense but very uneven (Figure 1). The number of pads impacted among those with continuous data records during the study period was 2335. Variables obtained from each impacted pad are kinetic energy in J/m^2^ (*E*_*c*_), number of impacts/m^2^ (*N*), ice mass in g/m^2^ (*M*), and maximum diameter in mm (*D*_max⁡_).

## 3. Spatial Variability of Hailfall

### 3.1. Methodology

Spatial analysis of measured variables for the hailpad networks of France was done using the Cressman method. This approach permits transformation of observational data to regularly spaced grid points [14]. We used a grid with resolution 0.02° × 0.025°. A grid of values was obtained by the Cressman interpolation for each variable and campaign. From these grids, a grid of average values was constructed for each variable over all 21 campaigns. 

Values from the 2335 pads impacted at 386 hailpad stations were used for interpolation of the variables across the study area, since there were no impacts on the remaining hailpads over the study period. With the objective of improved interpretation of the results, we performed correlation analysis between the four variables (listed at the end of Section 2). 

### 3.2. Results

Table 1 presents the Pearson correlations for the four variables measured with the hailpads. All correlations were statistically significant at the 0.01 level. Kinetic energy had a correlation coefficient of 0.926 with ice mass, and 0.715 with maximum diameter. These results are consistent since kinetic energy is a function of both measured diameter and ice mass of hailstones. Ice mass had a correlation of 0.701 with number of impacts. The number of impacts and maximum diameter gave the lowest correlation, 0.238. 

**Figure 2:**
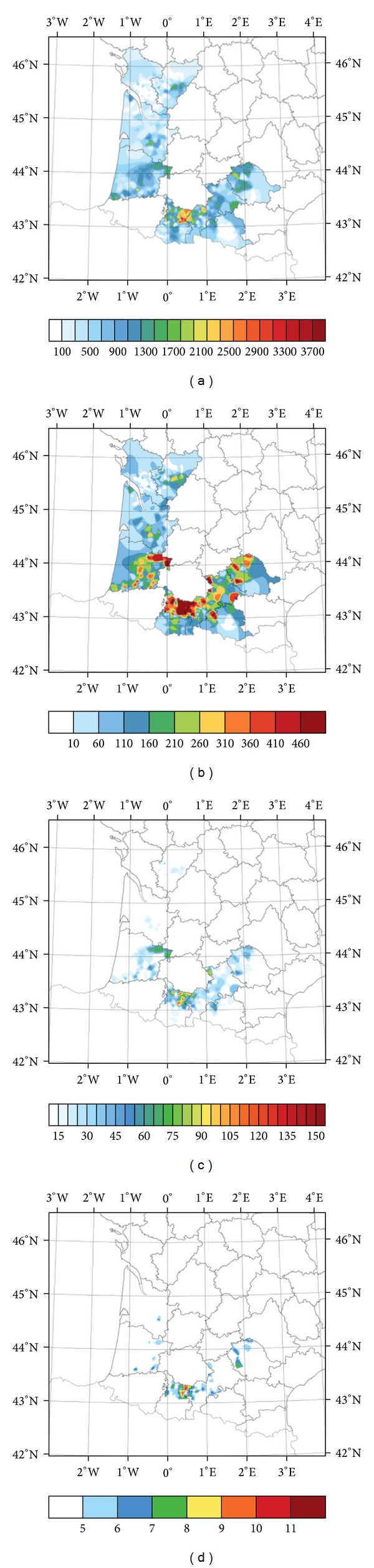
Spatial distribution of the average of variables: (a) number of impacts/m^2^, (b) ice mass (g/m^2^), (c) kinetic energy (J/m^2^), and (d) maximum diameter (mm) for the period of study between 1990 and 2010.

These correlations are reflected in the geographic distribution of average values of the variables from 1990 to 2010, shown in Figure 2. This figure shows that the central Pyrénées area had averages higher than all other variables, particularly in the northern Hautes-Pyrénées department. There, we found averages for the study period of more than 2500 impacts/m^2^, ice mass greater than 460 g/m^2^, kinetic energy exceeding 90 J/m^2^, and maximum diameter larger than 8 mm. This area coincides with one of the high hail hazard areas identified by Vinet [2]. In addition, as stated before, Berthet et al. [6] highlighted the Pyrénées area as one with the greatest frequency and intensity of hail, because of its proximity to the mountains and Ebro Valley of Spain.

The concentration of maximum values of the four variables in this area can be attributed to much stronger thermal convection, with heat sources at higher elevations, and probably to a dynamic effect that generates stronger winds at upper levels downwind of the barrier [12]. These winds are especially strong leeward of convective activity over mountain chains.

We compared the present results with those of earlier works, some of which are cited presently. Vinet [2] also indicated a greater disposition toward hail on the leeward side, where convective activity is increased; however, there are other geographic particularities, such as valleys and forests, which can be influential. Mezher et al. [15] found two large regions in Argentina with maximum hail frequency. One is in the provinces of Mendoza, La Pampa, and Córdoba, in the center and west of that country; the other extends along the meridional coast of Patagonia. In the first region, the maximum appears associated with increased convective activity supported by the mountains, which facilitate uplift of low-level humid air advected from the Atlantic Ocean. 

In central Macedonia, Sioutas et al. [1] observed greater hailfall in high-altitude areas close to the lee side of mountains and minimum values at lower elevations near the sea. 

Maximum hail frequencies in mountainous areas were also found by Etkin and Brun [16] in central British Columbia and Alberta of Canada, with highest values east of the Rockies. In China, Zhang et al. [17] found high average annual frequencies on the Tibetan Plateau and the Mount Qilian. Cepeda and Rodríguez [18] revealed that hail events in Bogota were spatially distributed, with greater density of occurrence in a north-south strip, parallel and congruent with the eastern mountains. Maximum occurrence density was La Candelaria, which is near those mountains. 

Marcelino et al. [19] studied hail events in the neighboring Brazilian state of Santa Catarina. There, they found a maximum regional frequency at São José do Cedro (near the Argentinian border), with a mean annual value of five events, which the authors attributed to regional topography. 

Regarding the spatial distribution of maximum diameter, Berthet et al. [6] showed that the number of stones with the largest diameters was between two and three times greater in continental areas than in those under the influence of the sea, likely owing to differences in intensity of convection and condensation nuclei.

 In the meridional Atlantic area, hail variable values tended to diminish, with minima in the areas of Charente and Charente-Maritime, in spite of a high density of hailpads (Figure 1). Storms are generally weak in the west of France [2]. The topography there is generally lower and the land flatter. The greatest separation between the freezing level and surface can explain the effect of fusion of hailstones during their fall through warm air, resulting in lower values for the variables.

 We found distinct characteristics of hail precipitation between the Atlantic and Midi-Pyrénées areas. Both are influenced by various air masses, as suggested by Berthet et al. [6]. On a small scale, however, there are other factors, such as orography, wind field changes, or differences, in concentration of condensation nuclei that affect hail climatology.

## 4. Temporal Variability of Hail Precipitation

### 4.1. Methodology

A monthly accumulation of impacted pads was used to obtain series of hail frequency. The data came from 443 stations that were in continuous use from 1990 to 2010. Locations of the hailpads are shown in Figure 1. Monthly trends of each hailpad were then calculated for May through September.

According to Conrad and Pollak [20], a numerical series representing variations in a climatological aspect can be defined as homogeneous if those variations are caused only by alterations in weather or climate. The non-parametric Kruskal-Wallis test was applied to each hailpad for verifying data series homogeneity. This test has been extensively used for the study of homogeneity in temporal series (e.g., [21, 22]). This test consists of ordering, from smallest to largest, values observed in k samples. A range is assigned, with 1 to the lowest value, 2 to the second, and so on. If there is a value that is equal in two or more cases, the average is assigned.

For analysis and detection of trends in the data series, linear regression can be used [6, 21, 23, 24]. The main advantage of this method is its simplicity. The main statistical parameter is slope, which indicates average change of a variable with time. Mohr and Kunz [25] applied linear regression to the calculation of trends in time series of hail-relevant convection parameters. To analyze the statistical significance of linear trends, the non-parametric Mann-Kendall test is used. Tabari and Hosseinzadeh Talaee [23] used Student's *t*-test with the same objective.

The present analysis of time series was carried out with the aforementioned Mann-Kendall test. This test is often applied in climatic studies to detect trends in data series, statistically determining if values are increasing or decreasing over a period. This test has weak sensitivity to sharp breaks, owing to the inhomogeneity of temporal series [23], and can use missing values. The null hypothesis *H*_0_ indicates nonexistence of a monotonic trend in a variable over time. 

This test is calculated by

(1) $$\begin{array}{c} S=\overset{n-1}{\underset{k=1}{\sum }}\;\overset{n}{\underset{j=k+1}{\sum }}\mathrm{sgn}\left(x_{j}-x_{k}\right), \end{array}$$

where *x*_*j*_ and *x*_*k*_ represent values of *x* in years *j* and *k*, respectively:

(2) $$\begin{array}{c} \mathrm{sgn}\left(x_{j}-x_{k}\right)=\{\begin{array}{cc} +1 & \text{if}\left(x_{j}-x_{k}\right)>0\\ 0 & \text{if}\left(x_{j}-x_{k}\right)=0\\ -1 & \text{if}\left(x_{j}-x_{k}\right)<0. \end{array} \end{array}$$

If the number of data in the temporal series is greater than or equal to ten, the normal approximation test is applied [26].

The variance of *S* is calculated as

(3) $$\begin{array}{c} \mathrm{Var}\left(S\right)=\frac{1}{18}\left[n\left(n-1\right)\left(2n+5\right)-\overset{q}{\underset{p=1}{\sum }}t_{p}\left(t_{p}-1\right)\left(2t_{p}+5\right)\right], \end{array}$$

where *q* is the number of tied groups and *t*_*p*_ is the number of data values in the *p*th group.

Thus, the statistic is computed as

(4) $$\begin{array}{c} Z=\{\begin{array}{cc} \frac{S-1}{\sqrt{\mathrm{Var}\left(S\right)}} & \text{if}\;\;S>0\\ 0 & \text{if}\;\;S=0\\ \frac{S+1}{\sqrt{\mathrm{Var}\left(S\right)}} & \text{if}\;\;S<0. \end{array} \end{array}$$

The statistic is determined by counting the number of positive and negative signs over the study period. A zero statistic indicates no change in trend over time, and the null hypothesis is accepted.

The *MAKESENS* application from the Finnish Meteorological Institute [26] was used to calculate trends with the Mann-Kendall test, as well as the statistical significance of these trends. The main limitation of that test occurs when there is serial correlation of the data in space and time. The existence of positive serial correlation increases the probability that the test detects a trend when none is present (false positives). This will cause rejection of the null hypothesis when it is valid in reality. A negative serial correlation diminishes the possibility of rejecting the null hypothesis [27]. To verify independence of the temporal series, the serial correlation coefficient *r*_*k*_ was calculated for a first-order autoregressive process:

(5) $$\begin{array}{c} r_{k}=\frac{\sum_{i=1}^{N-k}\left(x_{i}-\overset{-}{x}\right)\left(x_{i+k}-\overset{-}{x}\right)}{\sum_{i=1}^{N}{\left(x_{i}-\overset{-}{x}\right)}^{2}}. \end{array}$$

The null hypothesis is that there is no correlation between two consecutive observations (*H*_0_:  *r*_1_ = 0). If *r*_1_ is not significant at 5%, then the Mann-Kendall test is applied to the original series. Otherwise, before applying the test, pre-whitening is done as (*x*_2_ − *r*_1_ *x*_1_, *x*_3_ − *r*_1_ *x*_2_,…, *x*_*n*_ − *r*_1_ *x*_*n*−1_) [23]. This method was proposed by von Storch and Navarra [28] for replacing the original temporal series.

### 4.2. Discussion

#### 4.2.1. Trends in Frequency of Hail Occurrence

The trend in hail frequency was calculated for each station, for the time series from 1990 to 2010. The annual series of 346 stations were found homogeneous at significance level 0.05. Homogeneity could not be guaranteed for 40 hail stations, and these were not included in the study. Correlation was eliminated for 19 stations. There was no impact registered on 57 hailpads over the study period.

**Figure 3:**
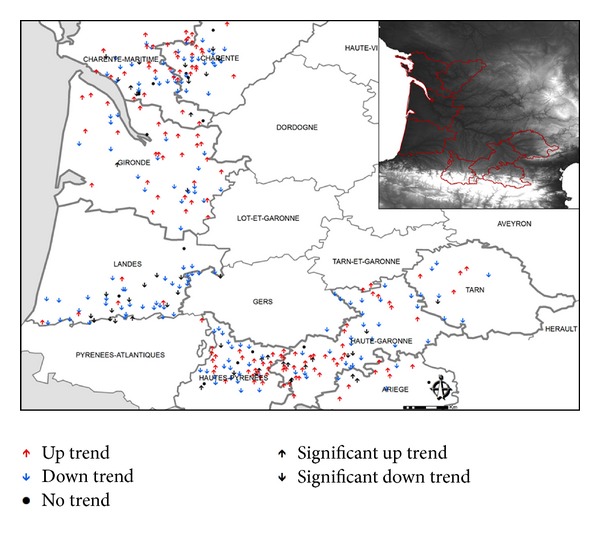
Geographical distribution of hailpads with upward, downward, and no trend from 1990 to 2010. The significant trends are shown in black.

Annual trends for the number of pads impacted over the entire study area are shown in Figure 3. Significant trends are shown with black arrows.

**Figure 4:**
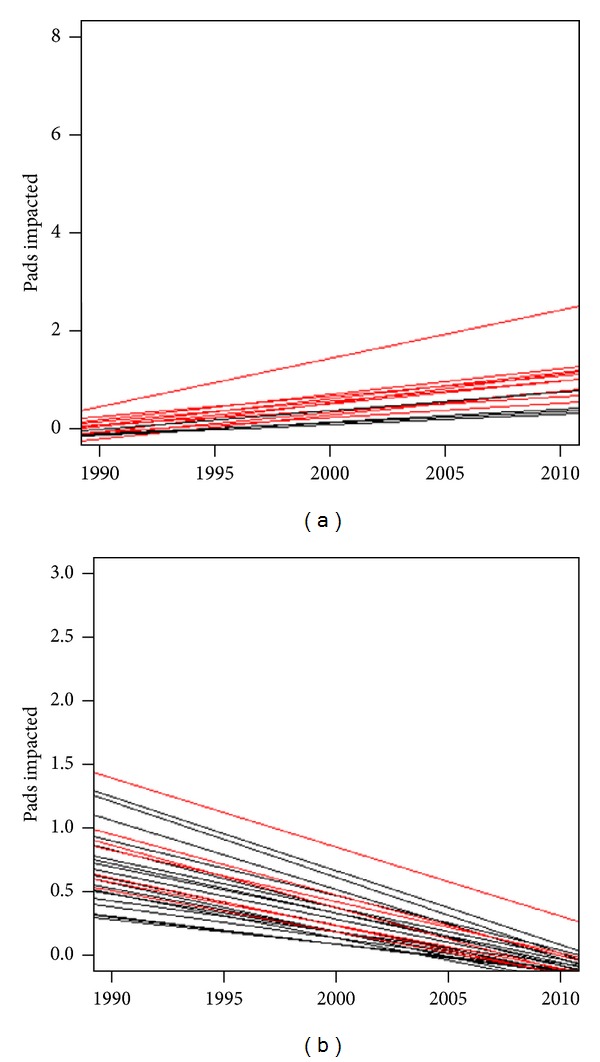
Linear trends of hailpad stations with significant trends. (a), upward trends; (b), downward trends. In black, trends of the Atlantic area; in red, trends of the Midi-Pyrénées area.

We found 154 hailpads with a positive trend and 177 with negative trend (Table 2). Only 15 stations did not show any type of trend. About 17% of the stations with negative trend were significant, compared to about 10% with positive trends (Figure 4). There were 25 stations with trends significant at the 0.1 level, 13 with negative trends and 12 with positive ones. There were 20 with trend significant at 0.05, 17 with a negative trend and 3 with positive one. There was one station with a positive trend at significance level 0.01, at Lasserre in the department of Ariège. 

If we consider the trends of hail frequency in both areas separately, as presented in Table 2, we find a predominant negative trend in the Atlantic area. There were 112 hailpads with this trend, compared to 74 with positive trend. In the Midi-Pyrénées area, the trend difference is less, with 80 stations having a positive trend and 65 with negative trend. Pads without any trend in the Atlantic area were twice those in the Midi-Pyrénées area. 

In the Atlantic area, there were 23 hailpads with a significant negative trend, with 12 at significance levels 0.05 and 11 at 0.1 (Figure 4). In contrast, in the Midi-Pyrénées area, there were a greater number of stations (12) with significant positive trend, among which was Lasserre. 

The hailpads with negative trend in the Atlantic area concentrated south of the large pine forest in Landes. In the Midi-Pyrénées, the stations with positive trends congregated between Tarbes and Saint-Gaudens. This area, with a greater number of stations with positive trends, is where values were higher for the four variables studied with the hailpads. The remaining stations had increased variability in spatial distribution of pads with positive and negative trends, indicating no apparent pattern.

The data reveal some opposite trends, even for relatively close hailpads (separated by only a few kilometers). This reflects that hailfall is a small-scale phenomenon, characterized by strong spatial and temporal variability.

It is essential to consider this small spatial scale of hailfall. Taking this into account with orography and other factors important to hail, such as updrafts facilitating particle growth, freezing levels, or frozen nuclei concentration, it seems obvious that their influences can also be minimized by increasing the quantity of hailpads in certain areas. 

One should also point out the influence of the predominance of certain air masses in the area. The Atlantic region is strongly affected by maritime conditions, while the Midi-Pyrénées region has continental and mountainous influences [3]. 

The variability of spatial distribution of climatic trends in hail frequency makes it difficult to interpolate a trend at regional scale by considering only statistically significant hailpad stations. This is because although there may be pads with significant trends at nearby stations during the same period, very small-scale factors can have strong influence [21]. Regional-scale studies in North America have indicated that long-term frequencies greatly varied over short distances, even in flat areas such as Illinois [9]. Giaiotti et al. [4] illustrated the heterogeneity of spatial frequency, even between relatively close areas on the plain of Friuli Venezia Giulia.

#### 4.2.2. Monthly Trends

Table 3 gives results of the Mann-Kendall test for the monthly series of hail occurrence frequency. In general, the monthly number of pads impacted had a decreasing trend, with greater magnitude in August, followed by June and July. The trends in May were predominantly positive, although there were only 15 stations more than those with negative trend. May also had the smallest number of stations with zero impacts over the 21 experimental campaigns. Dessens and Fraile [29] found that the total number of impacts in both areas was greater in this month, because of lower altitudes of the 0°C level.

In contrast, 267 stations were unaffected by hail in September, and in this month all hailpad stations showed some type of trend. Of the stations with positive trend in May, 7.95% showed statistical significance of 0.1, 0.05, or 0.01. In June, July, and August, a negative trend was dominant (especially in August), with 120 hailpads. There were 44 pads with positive trend, and 207 showed no impacts. In June, August, and September, none of the positive trends were significant, but 10 stations had a significant negative trend. Of the few stations impacted in September (about 119), trends were divided almost evenly, with positive trends at 60 stations and negative at 59. 

The trend was predominantly negative in both areas (Table 5). In the Atlantic area (Table 5), the number of stations with such a trend was always greater from June to August, from 45 to 74, and 59 in July. On the contrary, the greater quantity of stations with positive trend was in May, followed by July. The minimum was in August, followed by September. In the Midi-Pyrénées area (Table 5), the negative trend diminished with the progression of summer, from 65 stations in June to 46 in August. May and July again had the greatest number of stations with positive trend, with the least number in August. The stations with positive trend followed the same temporal pattern in both areas. The negative trends in June, July, and August showed completely opposite behavior.

The data appear to indicate a general trend toward hail occurrence early in the campaigns. This finding can be contextualized within the framework of global warming, especially with the increase of minimum temperatures. In May, there was an observed increase of 1.4°C in minimum temperature (although not significant) at Toulouse and Bordeaux stations, representing the Midi-Pyrénées and Atlantic areas, respectively [6].

Positive and negative monthly trends did not show any spatial pattern (data not shown).

## 5. Altitudinal Variability of Precipitation

The spatial distribution of the hailpads is shown by Figure 1, in which we see two areas of greater density. One is in the Atlantic area, which includes the Charente, Charente-Maritime, and Gironde stations, and the other is in the Midi-Pyrénées area, which includes Ariège, Hautes-Pyrénées, Haute-Garonne, and Tarn. These areas have hailpad networks with greater density of stations. This facilitates more detailed hailfall study, since there is a greater probability of recording hailfall events (not just large ones). Dessens and Fraile [29] pointed this out and recognized Cognac, Mont-de-Marsan, and Toulouse as areas with greater densities that would allow such detailed study.

 In the Midi-Pyrénées area, the concentration of hailpads with positive trend increased with proximity to the Pyrénées. This could be related to an increase in altitude, because precipitation often increases with altitude because of orographic effects. S. A. Changnon and D. Changnon [30] studied the number of days with hail in the United States using a database of 100 years. He revealed positive trends in a large part of the plains from Texas to North Dakota, including the central Rocky Mountains, and the coastal southeast area. In Northern Greece, strong hail variability over short distances and correlation with the topography has been noted. This was because maximum hail occurrence was at high altitudes near the leeward sides of mountain barriers, as mentioned previously [1]. On the plain of Friuli Venezia Giulia, Giaiotti et al. [4] found three areas of greater hail frequency, which was explained by interactions between cold fronts and the complex orography. 

**Figure 5:**
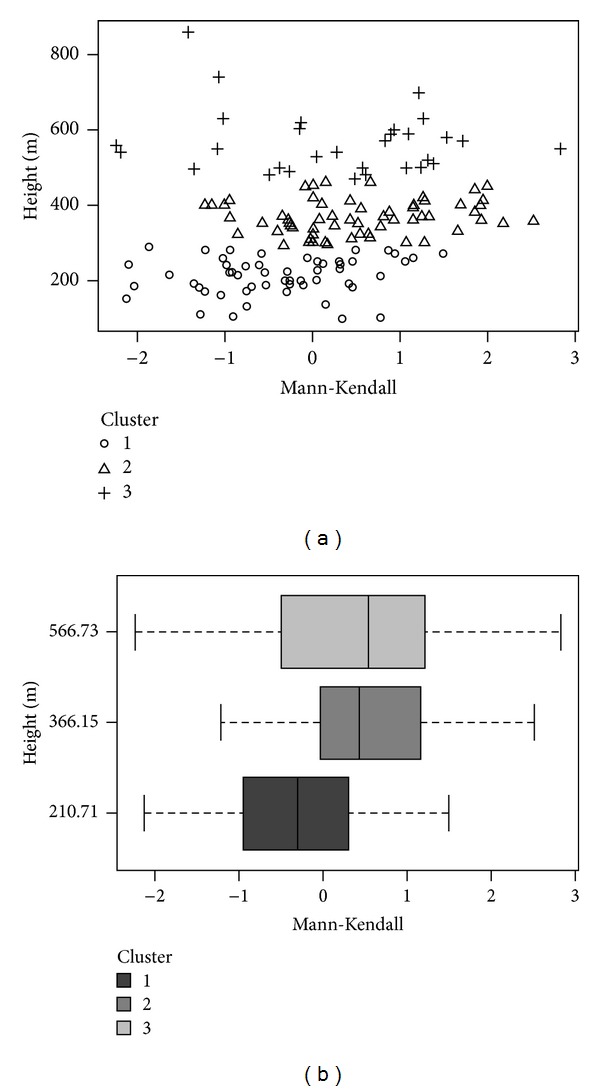
(a) Graphic representation of the Mann-Kendall test values for each one of the altitudes at which the hailpads are installed. (b) Boxplot of the three cluster resultants from the *k*-means cluster analysis. We can see the minimum and maximum values, the median and the first and third quartile for each cluster.

We made a first approximation with the representation of Mann-Kendall values (Table 4) for altitude corresponding to hailpad locations (Figure 5(a)). To determine variability with orography, a k-means cluster analysis was applied to values of the Mann-Kendall test and altitude, obtaining three clusters (Figure 5(b)). The third cluster includes hailpads at the highest altitudes, closer to mountains, with an ample range of values for the Mann-Kendall test. These values are both positive and negative. This cluster shows the greatest variance for both parameters (Table 6). The first cluster contains hailpads in the flattest departments, furthest from the Pyrénées, with trend values that are equally positive and negative. This cluster gives the smallest variance for the Mann-Kendall statistic (0.74), detecting a slight predominance of negative values. This predominance can be related to an increase of temperature. At the Pic du Midi observatory, mean annual temperature increased by 0.83°C from 1882 to 1970, while the minimum temperature increased by 2.11°C [24]. These changes increase the probability of hail melt before reaching the ground and thereby fewer impacts on the hailpads. This effect is more important for small hailstones [31].

The final cluster includes hailpads at altitudes intermediate to those of the other clusters. This cluster also showed positive and negative trends, but more positive ones.

In summary, the cluster analysis shows that spatial variability of the climatic trends is greater at higher-altitude stations. Consequently, to obtain representative values, it would be necessary to have a greater density of hailpads in these areas. Tapiador et al. [32] found similar results in an analysis of rain. He observed that differences of precipitation at kilometer scale were considerable, and they increased in mountainous areas because of the orographic influence. 

## 6. Spatial Variability of Hail Precipitation in Other Study Areas

The aforementioned results show that spatial variability of hail trends in France is extremely high. We wished to know if this variability is also high in other areas. To do so, we analyzed the Mediterranean area of southeast France and the province of Lérida in Spain. The southeast area of France corresponds with the departments of Gard, Vaucluse, Bouches du Rhône, and Drôme.

In Lérida, data were taken from 174 hailpads that were in use throughout all campaigns; among these, three did not register any impact. For southeast France, there were 97 pads, 36 of which showed no impact over a 10-year study period. We did trend analysis of corresponding annual series in both areas, following the previously mentioned methodology.

**Figure 6:**
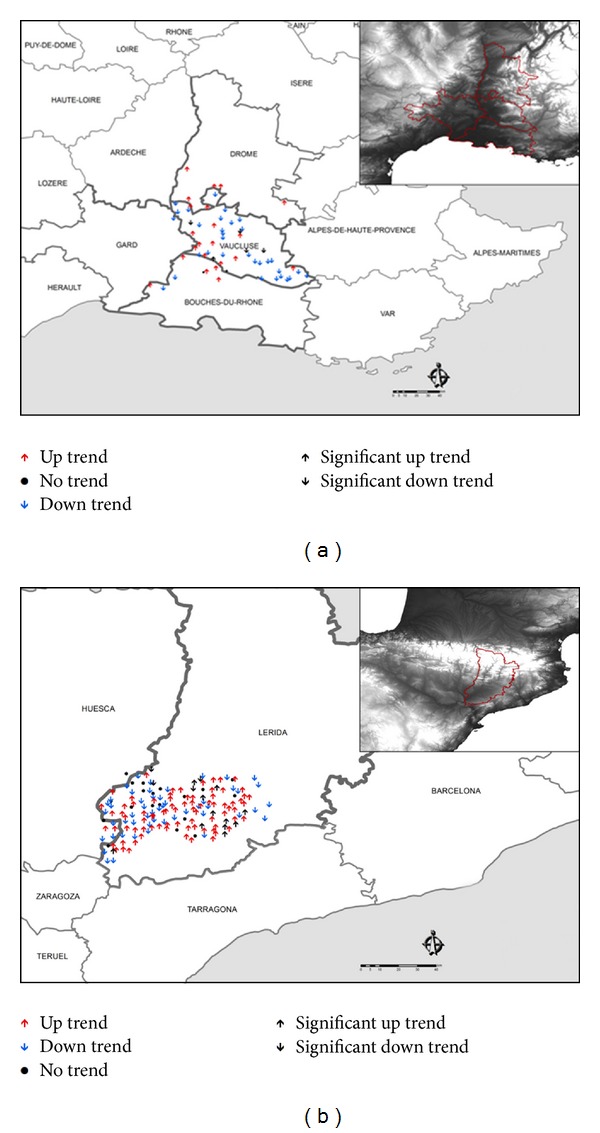
Geographical distribution of hailpads with upward, downward, and no trends, for the number of pads impacted from 2001 to 2010 in the Southeast of France (a), and from 2000 to 2011 in Lérida (Spain) (b).

In Figure 6, trends of hail frequency are shown for 2001–2010 in the French area and 2000–2011 in Spain.

The results from Southeast France show 23 hailpads with positive trend and 36 with a negative one (Table 7). In Lérida, there was a predominant positive trend, with 90 hailpads, versus 55 with a negative trend. Four of the stations with negative trend were statistically significant at 0.1; 9.9% of stations with positive trend were statistically significant, six at significance level 0.1, and the rest is at 0.05. In Southeast France, only one station had a significant trend at 0.1. Among stations with negative trend, three had significance level 0.1 and one had 0.05. There was again no geographic pattern discernible, which again highlights the aforementioned spatial variability.

## 7. Conclusions

In France, the spatial variability of hail climatic trends obtained from individual hailpads was extremely high. Similar results were found in Lérida (Spain) and Southeast France. Consequently, it was impossible to interpolate hail trends within the study areas. Accordingly, climatic studies should not use values interpolated from point data trends when validating or calibrating models, since those values are only representative of trends obtained from specific values. Moreover, when studying spatial variability, it is necessary to consider mesoscale factors, since they help explain the estimated trends. Even over a small area, there are opposite trends. This is possibly linked to storm paths, which cause hail on certain hailpads but not on nearby pads. This hypothesis could be tested in future study in greater depth. We found even greater variability with altitude. The variability of climatic trends in the number of pads impacted varies with altitude. The spatial variability of precipitation increases in areas at higher elevation. These results should be considered when designing networks or validating models. The Atlantic and Midi-Pyrénées areas showed different hail precipitation characteristics. The area between Tarbes and Saint-Gaudens had high average values of the variables. The study areas indicated opposite temporal variabilities during June, July, and August. May had the most hailpad impacts and a predominance of upward trends.

## Figures and Tables

**Table 1:** Correlations between hailfall parameters measured. Bold type shows significant correlations at 0.01 level.

	*N*	*M*	*E*_*c*_	*D*_max⁡_
*N*	1			
*M*	**0.701**	1		
*E*_*c*_	**0.447**	**0.926**	1	
*D*_max⁡_	**0.238**	**0.646**	**0.715**	1

*N*: total number of hailstones/m^2^.

*M*: total mass of hailstones, in g/m^2^.

*E*_*c*_: total kinetic energy of hailstones, in J/m^2^.

*D*_max⁡_: diameter of largest hailstones, in mm.

**Table 2:** Number of stations with positive and negative annual trends, and without trends.

	Up	Down	No trend
Atlantic	74	112	10
Midi-Pyrénées	80	65	5

**Table 3:** Number of stations with positive, negative, and no trends, along with number of stations without impacts (1990–2010). In parentheses are numbers of stations with significant trend.

	Up	Down	No trend	Zero impacts
May	151 (12)	136 (7)	17	82
June	79	110 (3)	4	193
July	94 (2)	113 (3)	16	163
August	44	120 (6)	15	207
September	60	59 (1)	0	267

**Table 4:** Number of stations with positive, negative, and no trends, for number of pads impacted from 1990 to 2010 in the areas with greatest density in Atlantic and Midi-Pyrénées areas.

	Up	Down	No trend
Atlantic	68	64	8
Midi-Pyrénées	78	64	5

**Table 5:** Number of stations with positive and negative monthly trends from 1990 to 2010, in Atlantic and Midi-Pyrénées areas.

	Atlantic		Midi-Pyrénées	
	Up	Down	Up	Down
May	83	76	68	60
June	38	45	41	65
July	44	59	50	54
August	11	74	33	46
September	20	28	40	31
	**196**	**282**	**232**	**256**

**Table 6:** Values from *k*-means cluster analysis for values of Mann-Kendall test, for annual series of number of hailpads impacted from 1990–2010, and altitude (m) of these pads.

Cluster	Parameter	*n*	Minimum	Maximum	Average	Variance
1	Mann-Kendall	56	−2.13	1.50	−0.38	0.74
	Height (m)		96	290	210,71	2607.15
2	Mann-Kendall	61	−1.24	2.53	0.5071	0.83
	Height (m)		291	460	366.15	2085.06
3	Mann-Kendall	30	−2.25	2.84	0.2480	1.49
	Height (m)		470	860	566.73	7249.44

**Table 7:** Number of stations with positive, negative, and no annual trends, for number of pads impacted from 2001 to 2010 in Southeast France and Lérida (Spain).

	Up	Down	No trend
Southeast (France)	23	36	2
Lérida (Spain)	90	55	19
